# Hub Genes, Diagnostic Model, and Predicted Drugs Related to Iron Metabolism in Alzheimer's Disease

**DOI:** 10.3389/fnagi.2022.949083

**Published:** 2022-07-07

**Authors:** Xuefeng Gu, Donglin Lai, Shuang Liu, Kaijie Chen, Peng Zhang, Bing Chen, Gang Huang, Xiaoqin Cheng, Changlian Lu

**Affiliations:** ^1^Shanghai Key Laboratory of Molecular Imaging, Zhoupu Hospital, Shanghai University of Medicine and Health Sciences, Shanghai, China; ^2^School of Pharmacy, Shanghai University of Medicine and Health Sciences, Shanghai, China; ^3^School of Health Science and Engineering, University of Shanghai for Science and Technology, Shanghai, China; ^4^School of Clinical Medicine, Shanghai University of Medicine and Health Sciences, Shanghai, China; ^5^Department of Neurosurgery, Affiliated Hospital of Guangdong Medical University, Zhanjiang, China; ^6^Department of Neurology, Zhongshan Hospital, Fudan University, Shanghai, China

**Keywords:** Alzheimer's disease, iron metabolism, hub gene, diagnostic, drug, immune

## Abstract

Alzheimer's disease (AD), the most common neurodegenerative disease, remains unclear in terms of its underlying causative genes and effective therapeutic approaches. Meanwhile, abnormalities in iron metabolism have been demonstrated in patients and mouse models with AD. Therefore, this study sought to find hub genes based on iron metabolism that can influence the diagnosis and treatment of AD. First, gene expression profiles were downloaded from the GEO database, including non-demented (ND) controls and AD samples. Fourteen iron metabolism-related gene sets were downloaded from the MSigDB database, yielding 520 iron metabolism-related genes. The final nine hub genes associated with iron metabolism and AD were obtained by differential analysis and WGCNA in brain tissue samples from GSE132903. GO analysis revealed that these genes were mainly involved in two major biological processes, autophagy and iron metabolism. Through stepwise regression and logistic regression analyses, we selected four of these genes to construct a diagnostic model of AD. The model was validated in blood samples from GSE63061 and GSE85426, and the AUC values showed that the model had a relatively good diagnostic performance. In addition, the immune cell infiltration of the samples and the correlation of different immune factors with these hub genes were further explored. The results suggested that these genes may also play an important role in immunity to AD. Finally, eight drugs targeting these nine hub genes were retrieved from the DrugBank database, some of which were shown to be useful for the treatment of AD or other concomitant conditions, such as insomnia and agitation. In conclusion, this model is expected to guide the diagnosis of patients with AD by detecting the expression of several genes in the blood. These hub genes may also assist in understanding the development and drug treatment of AD.

## Introduction

Alzheimer's disease (AD), the most common form of dementia, is a neurodegenerative disease associated with aging. According to a report in 2020, there are an estimated 5.8 million people aged 65 and older with AD in the United States, and this number could increase to 13.8 million by the middle of this century (Zhang T. et al., 2021). Typical AD occurs after the age of 65 years, and <5% of AD cases occur earlier in life, among which 1–2% occur in familial clusters (Long and Holtzman, 2019). The pathology of this disease is characterized by amyloid plaques comprised of amyloid-β (Aβ) peptides and neurofibrillary tangles (NFTs) containing hyperphosphorylated tau proteins (DeTure and Dickson, 2019). Various drugs available to treat AD, such as donepezil, galantamine, memantine, rivastigmine, and aducanumab (also known as Aduhelm), are not very effective (Barthold et al., 2020; Mullard, 2021). Therefore, finding new effective molecules to improve the diagnosis and treatment of AD is urgent.

As the second most abundant metal on the Earth's crust after aluminum, iron is one of the trace metals essential for human beings. Iron is an important component of hemoglobin, which is involved in oxygen transport. Iron is also involved in the metabolism of catecholamine neurotransmitters and the formation of myelin sheaths in the nervous system (Thirupathi and Chang, 2019; Peng et al., 2021). Iron homeostasis is maintained by a variety of complex mechanisms, such as iron regulatory proteins and hepcidin (Pantopoulos et al., 2012). Disruption of this homeostasis leads to excessive accumulation of intracellular iron, which can damage DNA, proteins, and lipids through the production of oxidative stress and free radicals (Ward et al., 2014). Abnormal iron accumulation has been observed in different brain regions of patients with AD (Mills et al., 2010; Apostolakis and Kypraiou, 2017; Lee and Lee, 2019). In AD, excessive accumulation of iron in the brain aggravates amyloid protein deposition and tau protein hyperphosphorylation, which leads to neuronal damage and cognitive impairment (Gong et al., 2019; Yan and Zhang, 2019; Choi et al., 2021; Peng et al., 2021). Iron can also directly induce oxidative damage to neurons (Thirupathi and Chang, 2019). In conclusion, there is a non-negligible relationship between iron metabolism and AD.

**Figure 1:**
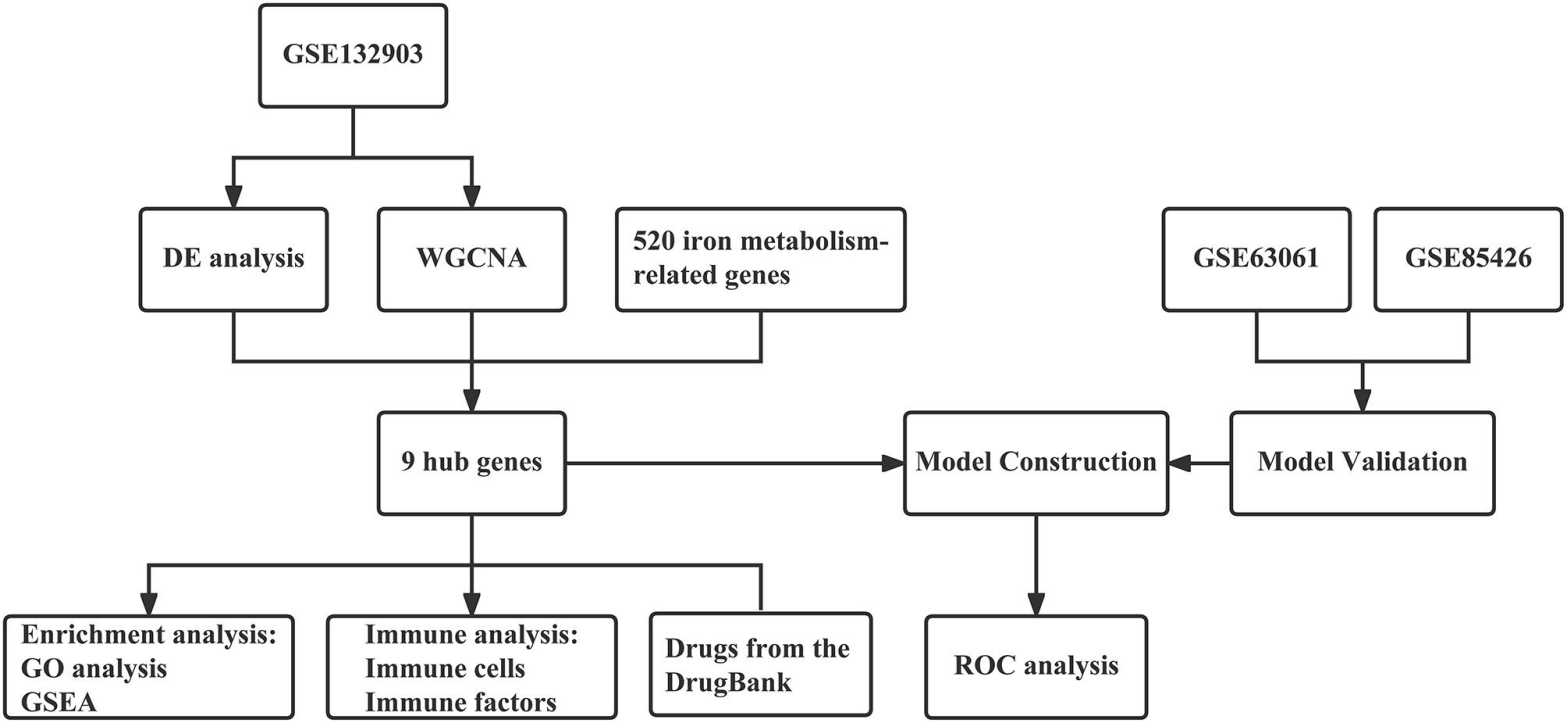
The workflow of the analyses.

Based on abundant public resources and bioinformatics methods, this study identified nine hub genes associated with iron metabolism and AD by differential analysis and WGCNA of GSE132903. GO and GSEA analyses were applied to further investigate the biological processes and pathways. Stepwise regression and logistic regression analyses were performed to screen four of them and construct a diagnostic model for AD, which was validated in two other blood sample datasets, GSE63061 and GSE85426. The AUC values indicated that this model has relatively good diagnostic performance and thus had a potential application in the clinical diagnosis of AD. Neuroinflammation, an important pathological feature of AD, is closely related to immunity. Therefore, we further explored the immune infiltration of these samples and the correlation of different immune factors with these hub genes. In addition, eight drugs targeting these hub genes were retrieved from the DrugBank database, which has implications for the pharmacological treatment of AD. The workflow of this study is shown in Figure 1.

## Materials and Methods

### Data Acquisition

Gene expression data were obtained from the NCBI Gene Expression Omnibus public database (GEO). GSE132903 contained RNA expression data annotated by GPL10558 in the middle temporal gyrus, which included 97 AD samples and 98 ND controls. Samples from two datasets GSE63061 and GSE85426 were extracted from peripheral blood. GSE63061 annotated by GPL10558 included 139 AD samples and 134 ND samples, and GSE85426 annotated by GPL14550 included 90 AD samples and 90 ND samples.

### Differential Expression Analysis

Differential expression analysis of AD and ND samples was performed using the “limma” package in R software. Genes with *p*_adj < 0.01 and abs(logFC) > 0.585 were considered as DEGs. Heat maps and volcano plots of DEGs were created using the “pheatmap” and “ggplot2” packages.

### Iron Metabolism-Related Genes

Fourteen iron metabolism-related gene sets were extracted from the Molecular Signatures Database v7.5.1 (MSigDB; Liberzon et al., 2015), including GOBP_CELLULAR_IRON_ION_HOMEOSTASIS, GOBP_HEME_METABOLIC_PROCESS, GOBP_IRON_COORDINATION_ENTITY_TRANSPORT, GOBP_IRON_ION_HOMEOSTASIS, GOBP_IRON_ION_IMPORT_ACROSS_PLASMA_MEMBRANE, GOBP_IRON_ION_TRANSPORT, GOBP_RESPONSE_TO_IRON_ION, GOMF_2_IRON_2_SULFUR_CLUSTER_BINDING, GOMF_4_IRON_4_SULFUR_CLUSTER_BINDING, GOMF_IRON_ION_BINDING, HALLMARK_HEME_METABOLISM, HEME_BIOSYNTHETIC_PROCESS, MODULE_540, and REACTOME_IRON_UPTAKE_AND_TRANSPORT (Mou et al., 2020). After removing the overlapping genes, the gene sets associated with iron metabolism contained 520 genes (Supplementary Table 1).

### Weighted Correlation Network Analysis (WGCNA)

To explore the co-expression relationships among the genes and the relationship between the genes and the phenotypes, a gene co-expression network was constructed using the “WGCNA” package in R software (Langfelder and Horvath, 2008). Based on the cluster trees, abnormal samples were removed. The top 5,000 genes with a median absolute deviation (MAD) > 1 were retained. The correlation coefficient between each gene pair was calculated to construct a similarity matrix. To ensure the construction of a scale-free network, a suitable soft threshold was chosen to transform the similarity matrix into an adjacency matrix. Subsequently, a topological overlap matrix (TOM) was created to measure the mean network connectivity of each gene. Based on the relevant parameters of the blockwiseModules function, such as minModuleSize and mergeCutHeight, genes with similar expression profiles were grouped into different modules using the dynamic tree cutting method. Each module was depicted in a different color, where the genes in gray modules represented genes that cannot be assigned to any module. The gene expression profile of each module was represented by the first principal component called the module eigengene (ME). MEs were used to assess the association between the modules and phenotypes. The module with the highest absolute value of the correlation coefficient was identified as the key module for further analysis. Module membership (MM) is the correlation coefficient between the expression value of a gene and the ME of a module, representing the correlation between this gene and this module. Gene significance (GS) was the correlation coefficient between the expression value of a gene and a phenotype, representing the association between genes and phenotypes.

### Identification of Hub Genes

To obtain hub genes associated with both iron metabolism and AD, the intersection of DEGs, genes obtained by WGCNA, and genes in the iron metabolism gene sets were taken using the “VennDiagram” package in R software. Differences in the expressions of hub genes between the AD and ND samples were represented by violin plots. The hypothesis tests used were the *t*-test and the Mann-Whitney *U*-test. The former was used if the data conformed to a normal distribution, and the latter if not. Significance was defined as *p* < 0.05.

### Enrichment Analysis

To investigate the biological mechanisms of the hub genes affecting AD, functional enrichment analyses were conducted. We first analyzed the biological processes (BP) of Gene Ontology (GO) in which these genes are involved, and the final results were presented in a chord diagram using the “GOplot” package in R software. Next, the respective functions of each gene were revealed by Gene Set Enrichment Analysis (GSEA). Samples were distinguished into two groups based on median values of hub gene expression levels, including the low expression group and the high expression group. Genes were sorted by logFC from the highest to the lowest, and the background gene sets were downloaded from MSigDB (Liberzon et al., 2015). The final results were presented using the “enrichplot” package in R software. All these analyses were conducted using the “clusterProfiler” package in R software, and the screening condition was *p*_adj <0.05.

### Logistic Regression Model

Logistic regression is a generalized linear regression analysis model that can be used for the automatic diagnosis of diseases. In this study, logistic regression with two response variables was used, representing the AD sample when the response variable was 1 and the ND sample when it was 0. Stepwise regression analysis was first used to eliminate factors that were not significant for the response variable, and only those that were significant were retained to simplify the model. The stepwise regression iteratively added or removed variables from the model until the statistical value of Akaike information criterion (AIC) was minimized. Afterward, logistic regression was used to fit the relationship between these significant factors and the response variable. Finally, the diagnostic efficacy of the model was evaluated using receiver operating characteristic curves (ROCs) and the area under the ROC curve (Coat et al., 2015; Lai et al., 2021). These analyses were performed with the “stats” and “pROC” packages in R software.

### Immune Infiltration and Immune-Related Factors

Immune cell infiltration in the microenvironment was assessed using CIBERSORT, which contains 547 biomarkers and 22 human immune cells, including plasma, B cell, T cell, and myeloid cell subpopulations. The tool is based on the linear support vector regression principle for deconvolution analysis of the expression matrix of immune cells. This study used expression data from GSE132903 and quantified the relative proportions of the 22 immune cells in each sample. In addition, Spearman correlation analysis was performed between hub genes and immune infiltration, and immune factors. This analysis was performed using the “psych” package in R software, and the results were displayed as heatmaps. Different immune factors were downloaded from the TISIDB database (Ru et al., 2019), including 24 immunoinhibitors, 45 immunostimulators, and 41 chemokines (Supplementary Table 2).

### Drugs From the DrugBank

Drugs targeting hub genes were retrieved from the DrugBank database. The DrugBank database is a cheminformatics and bioinformatics repository containing detailed information on drugs and their targets. The database collects more than 7,800 drugs, including nutraceuticals, experimental drugs, FDA-approved small-molecule drugs, and FDA-approved biotech drugs (Wishart et al., 2018). The DrugBank also has a large collection of SNP drugs useful for pharmacogenomic studies.

### Statistical Analysis

All analyses were performed in R software. The *t*-test and Mann–Whitney *U*-test were selected according to whether the data conformed to a normal distribution. Significance was usually defined as *p* < 0.05.

## Results

### Identification of Hub Genes Associated With AD and Iron Metabolism

**Figure 2:**
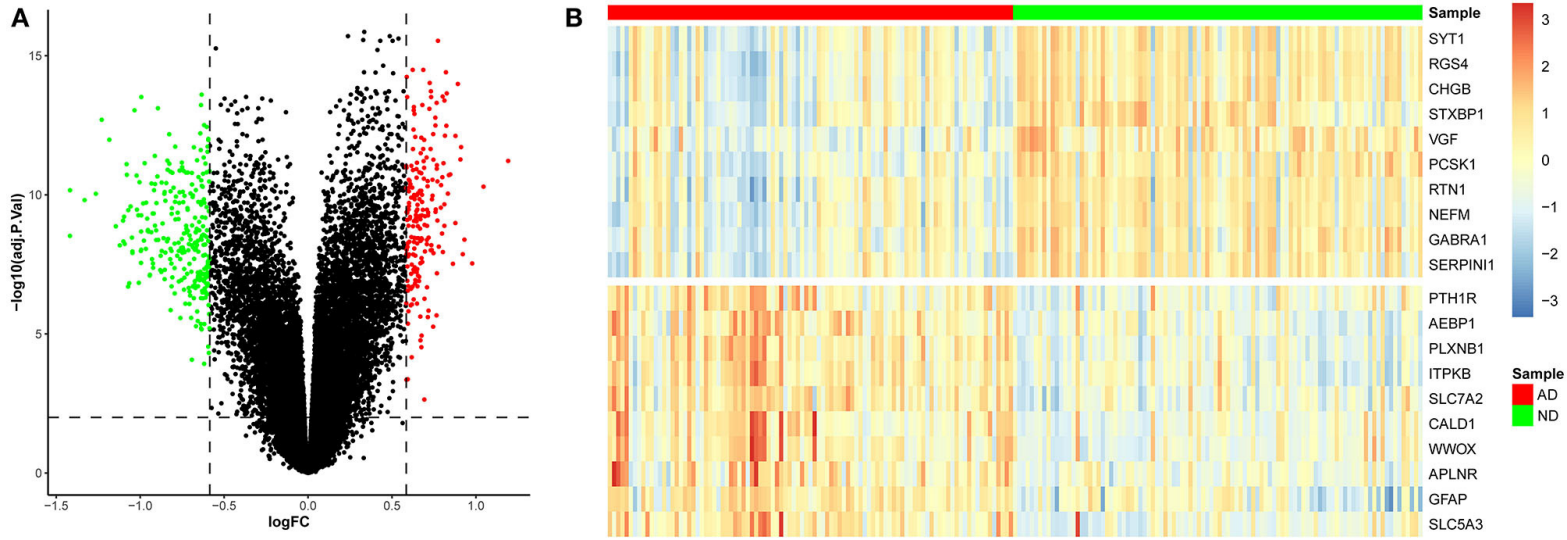
Differentially expressed genes between AD and ND samples. **(A)** Red genes represent significantly high expression in AD, green genes represent significantly high expression in ND, and gray genes indicate insignificant changes. **(B)** The heatmap shows the top 20 genes significantly highly expressed in AD or ND samples.

**Figure 3:**
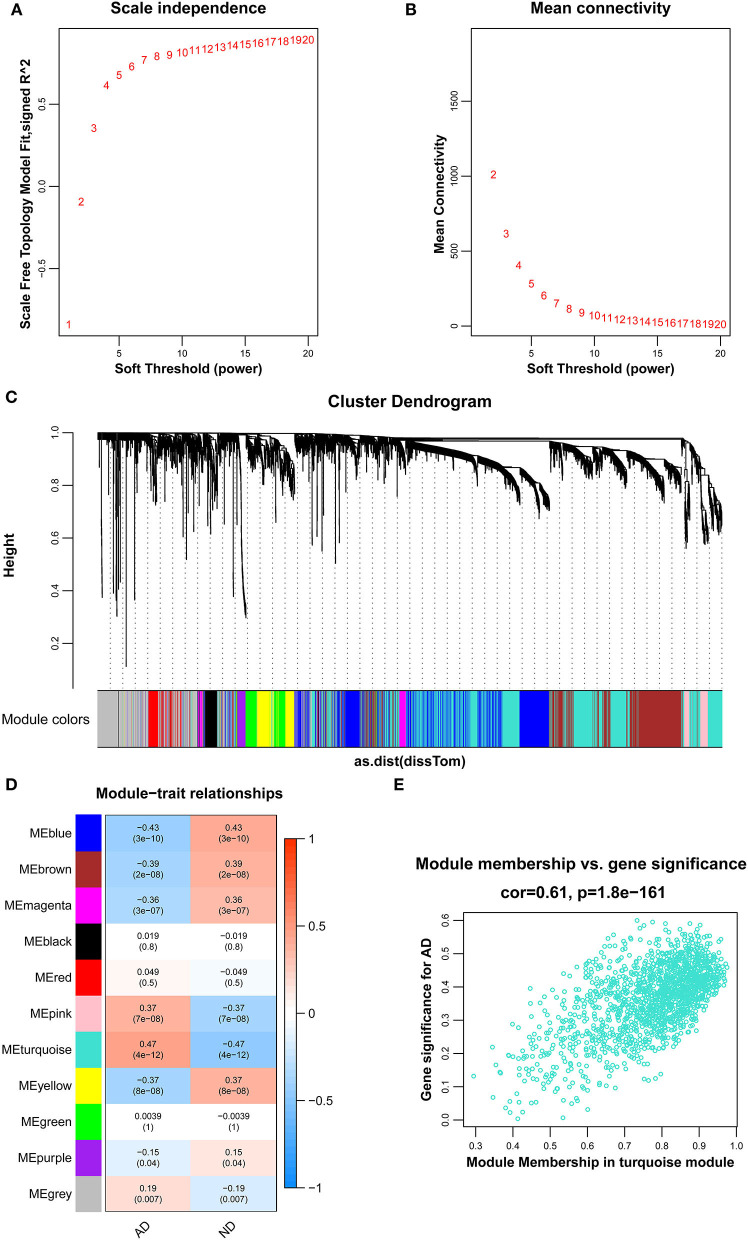
Results of the WGCNA. **(A)** The corresponding scale-free topological model fit indices at different soft threshold powers. **(B)** The corresponding mean connectivity values at different soft threshold powers. **(C)** Cluster dendrogram of genes. **(D)** Correlations between different modules and clinical traits. Red represents a positive correlation, and blue represents a negative correlation. **(E)** Correlation of module membership and gene significance in the turquoise module.

**Figure 4:**
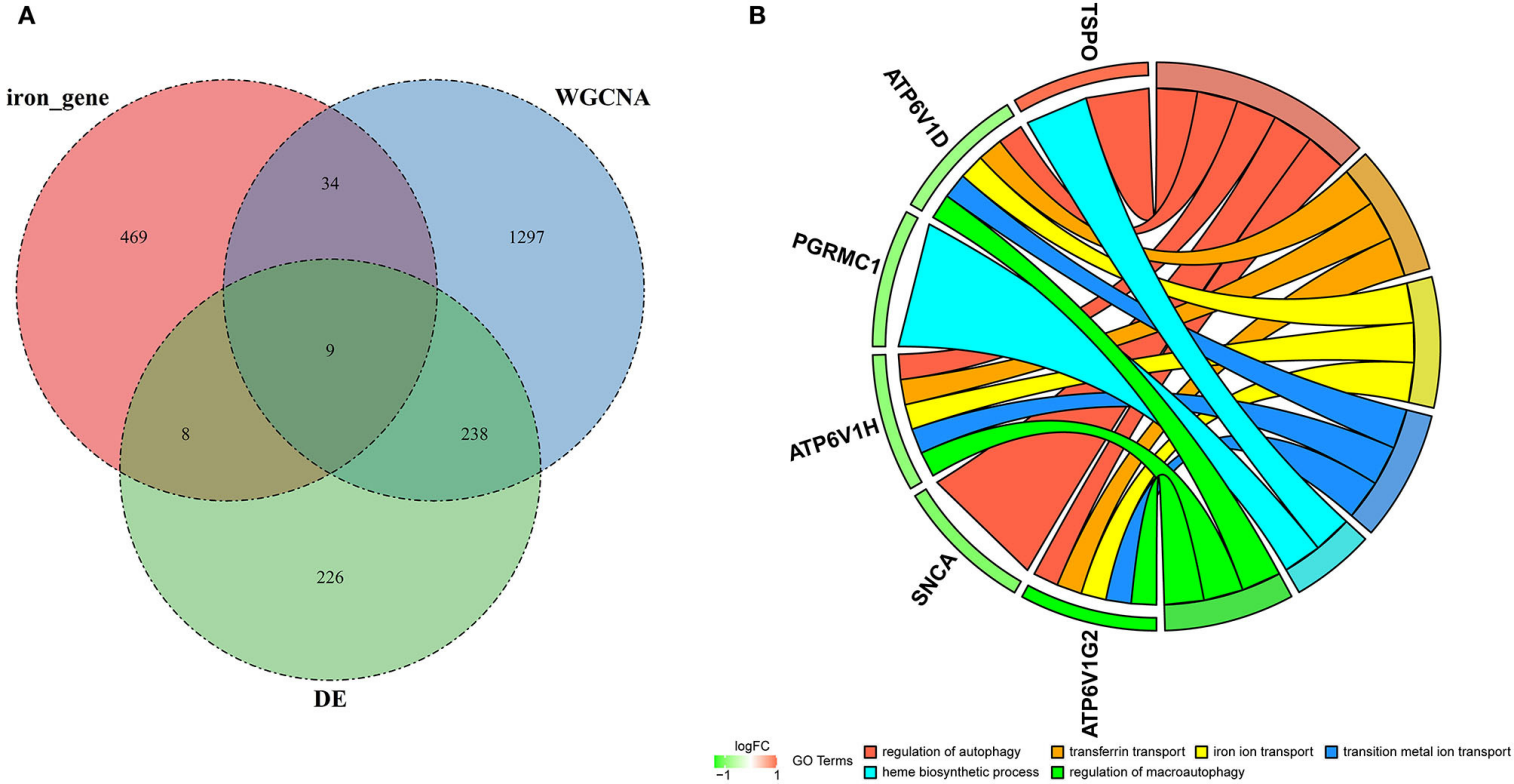
Hub genes and GO analysis. **(A)** Nine hub genes were obtained by taking the intersections of the DEGs, MEturquoise module genes of the WGCNA, and iron metabolism-related genes. **(B)** Biological processes in which the hub genes were involved.

**Figure 5:**
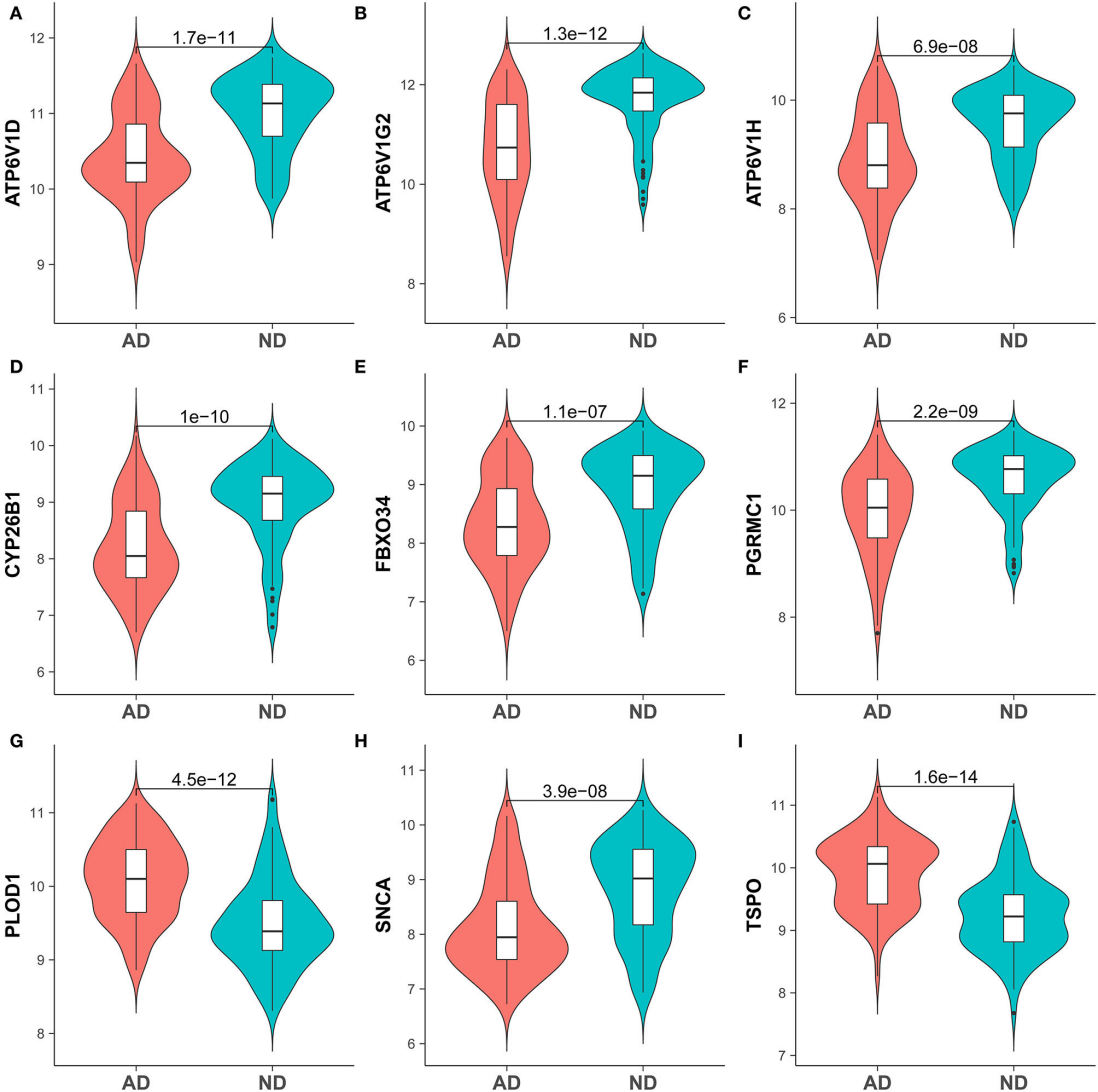
Expression of hub genes in the AD and ND groups of brain tissue samples GSE132903. **(A)** ATP6V1D, **(B)** ATP6V1G2, **(C)** ATP6V1H, **(D)** CYP26B1, **(E)** FBXO34, **(F)** PGRMC1, **(G)** PLOD1, **(H)** SNCA, and **(I)** TSPO.

To identify genes related to AD, we first obtained 481 differentially expressed genes from GSE132903 with the screening conditions “*p*_adj <0.01 and abs(logFC) > 0.585” (Supplementary Table 3). These DEGs were presented in a volcano plot (Figure 2A). A heatmap of the top 20 differentially expressed genes was plotted (Figure 2B). After removing abnormal samples and filtering the genes, the expression profiles of 5,000 genes and 195 samples were extracted from GSE132903 and used for the construction of a weighted gene co-expression network. When the soft threshold power was set to 14, the scale independence reached 0.858 and the average connection value was 30.124 (Figures 3A,B). When the cut height was set to 0.25 and the minimum module size was set to 50, 11 different co-expression modules were obtained by dynamic tree cutting (Figure 3C). Then, correlation analyses of each module with clinical traits were performed. The MEturquoise module had the highest positive correlation with AD (*r* = 0.47, *p* = 4e−12), while the MEblue module had the highest negative correlation with AD (*r* = −0.43, *p* = 3e−10; Figure 3D). Here, the MEturquoise module, containing 1,578 genes with the largest absolute value of correlation coefficient, was selected for further analysis. Additionally, correlation analysis between MM and GS showed that these genes were highly correlated with both module and phenotype (cor = 0.61, *p* = 1.8e−161; Figure 3E). Nine hub genes associated with iron metabolism and AD were obtained by taking the intersections of 481 DEs, 1,578 MEturquoise module genes, and 520 iron metabolism-related genes from 14 iron metabolism-related gene sets (Figure 4A). Violin plots showed that *TSPO* and *PLOD1* were highly expressed in AD, and the other seven genes were expressed at lower levels than in the ND group in GSE132903 (Figure 5). In addition, the expression of these genes in blood samples from GSE63061 and GSE85426 is shown in Supplementary Figures 1, 2.

### Biological Processes and Pathways Enriched for the Hub Genes

**Figure 6:**
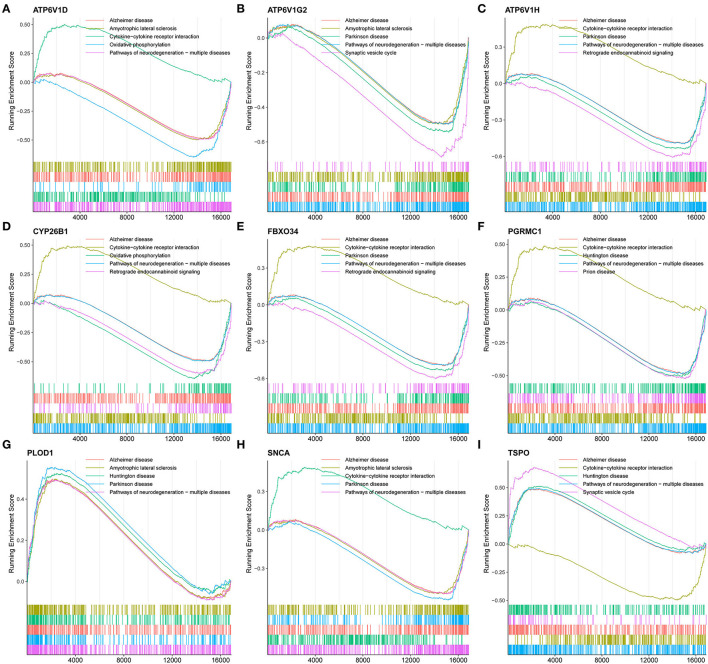
GSEA revealed the enriched pathways of the hub genes. **(A)** ATP6V1D, **(B)** ATP6V1G2, **(C)** ATP6V1H, **(D)** CYP26B1, **(E)** FBXO34, **(F)** PGRMC1, **(G)** PLOD1, **(H)** SNCA, and **(I)** TSPO.

To understand the potential biological roles of these genes, enrichment analyses were performed. GO analysis revealed that six of the nine genes were involved in autophagy-related biological processes, including regulation of autophagy and macroautophagy, and iron metabolism-related biological processes, including transferrin transport, iron ion transport, transition metal ion transport, and heme biosynthetic process (Figure 4B). GSEA of these genes has shown that they are associated with several neurodegenerative diseases (AD, amyotrophic lateral sclerosis, Parkinson's disease, Prion disease, and Huntington's disease), neurological related pathways (the synaptic vesicle cycle, retrograde endocannabinoid signaling, retrograde endocannabinoid signaling, and pathway of neurodegeneration), and other pathways (cytokine–cytokine receptor interaction and oxidative phosphorylation; Figure 6).

### Construction and Blood Validation of a Diagnostic Model

**Figure 7:**
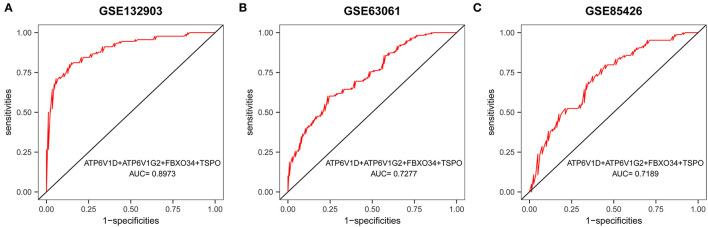
ROC curves and corresponding AUC values for the three expression cohorts. **(A)** Brain tissue samples from GSE132903. **(B)** Blood samples from GSE63061. **(C)** Blood samples from GSE85426.

A multigene prediction model was constructed by a logistic regression algorithm based on GSE132903. Using stepwise regression analysis, four of these nine genes, including *ATP6V1D, ATP6V1G2, FBXO34*, and *TSPO*, were selected to obtain the best model. The results showed that the predictive model constructed from these four genes had good diagnostic performance, with an AUC of 0.8973 (Figure 7A). The model was then further validated in blood samples. The AUCs of the models in GSE63061 and GSE85426 were relatively high, 0.7277 and 0.7189, respectively (Figures 7B,C). Brain tissue samples tended to be more representative of AD pathology than blood samples, which may explain the better diagnostic performance of the former. However, it was difficult to obtain brain tissue *in vivo*. The good results in blood samples suggested that this model has a certain guiding significance for the diagnosis of patients with AD in clinical applications.

### Immune Infiltration and Immune-Related Factors

**Figure 8:**
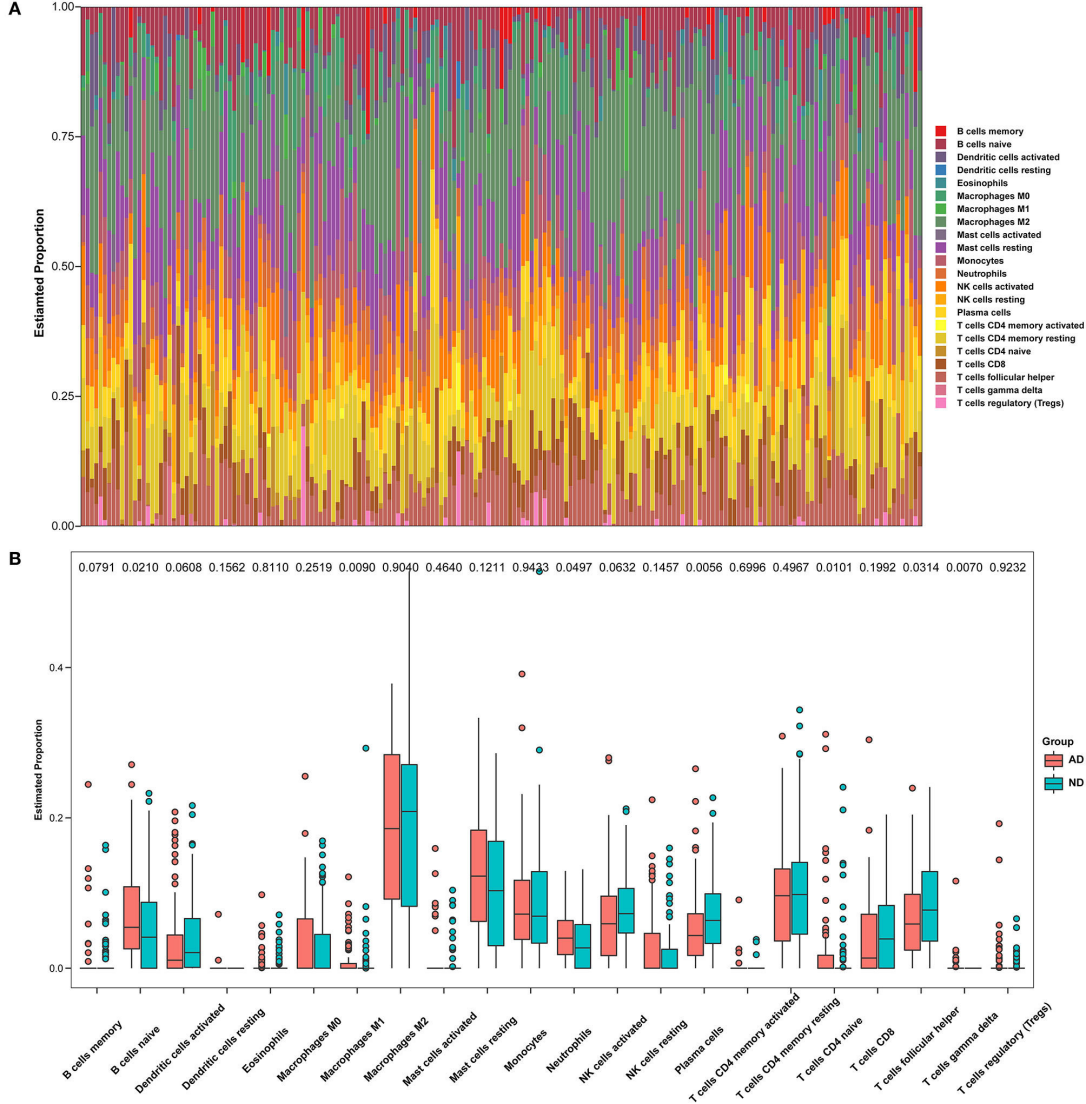
Immune infiltration between AD and ND samples. **(A)** The relative percentage of 22 immune cells in each sample. **(B)** Differences in immune infiltration between AD and ND samples.

**Figure 9:**
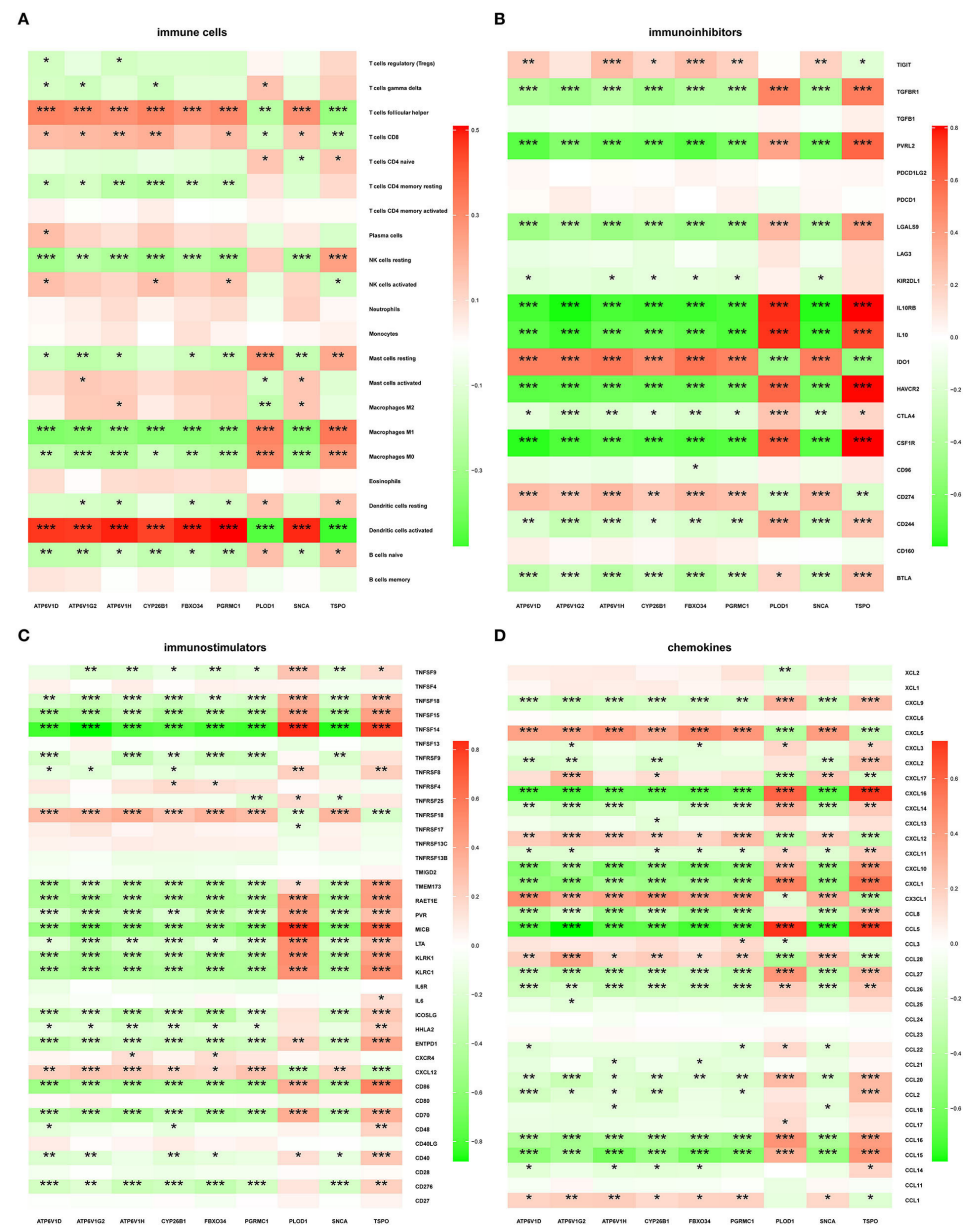
Correlation of hub genes with immune cells and different immune factors. **(A)** Immune cells, **(B)** immunoinhibitors, **(C)** immunostimulators, and **(D)** chemokines.

The microenvironment consists of immune cells, extracellular matrix, inflammatory factors, and various growth factors that have an important impact on the clinical therapeutic sensitivity and disease diagnosis. In this study, the proportion of 22 immune cells in 97 AD samples and 98 ND samples was estimated by the CIBERSORT algorithm, which can be seen in the histogram (Figure 8A). The immune cell infiltration of AD and ND samples was compared in a boxplot (Figure 8B). The results showed that the AD group had significantly higher proportions of naive B cells (*p* = 0.0210), M1 macrophages (*p* = 0.0090), neutrophils (*p* = 0.0497), CD4 naive T cells (*p* = 0.0101), and gamma delta T cells (*p* = 0.0070), and lower proportions of plasma cells (*p* = 0.0056) and follicular helper T cells (*p* = 0.0314) than the ND group. Next, the relationship between the hub genes and immune infiltration, and immune factors was analyzed. *PLOD1* and *TSPO* were significantly negatively associated with follicular helper T cells, CD8 T cells, and activated dendritic cells, and positively associated with resting NK cells, M1 macrophages, M0 macrophages, and naive B cells, while the opposite was true for the other downregulated genes (Figure 9A). The correlation heatmaps showed a significant correlation between the hub genes and most immune factors, including immunoinhibitors, immunostimulators, and chemokines (Figures 9B–D). These results suggested that the hub genes may play an important role in the immune microenvironment.

### Drugs From the DrugBank

**Figure 10:**
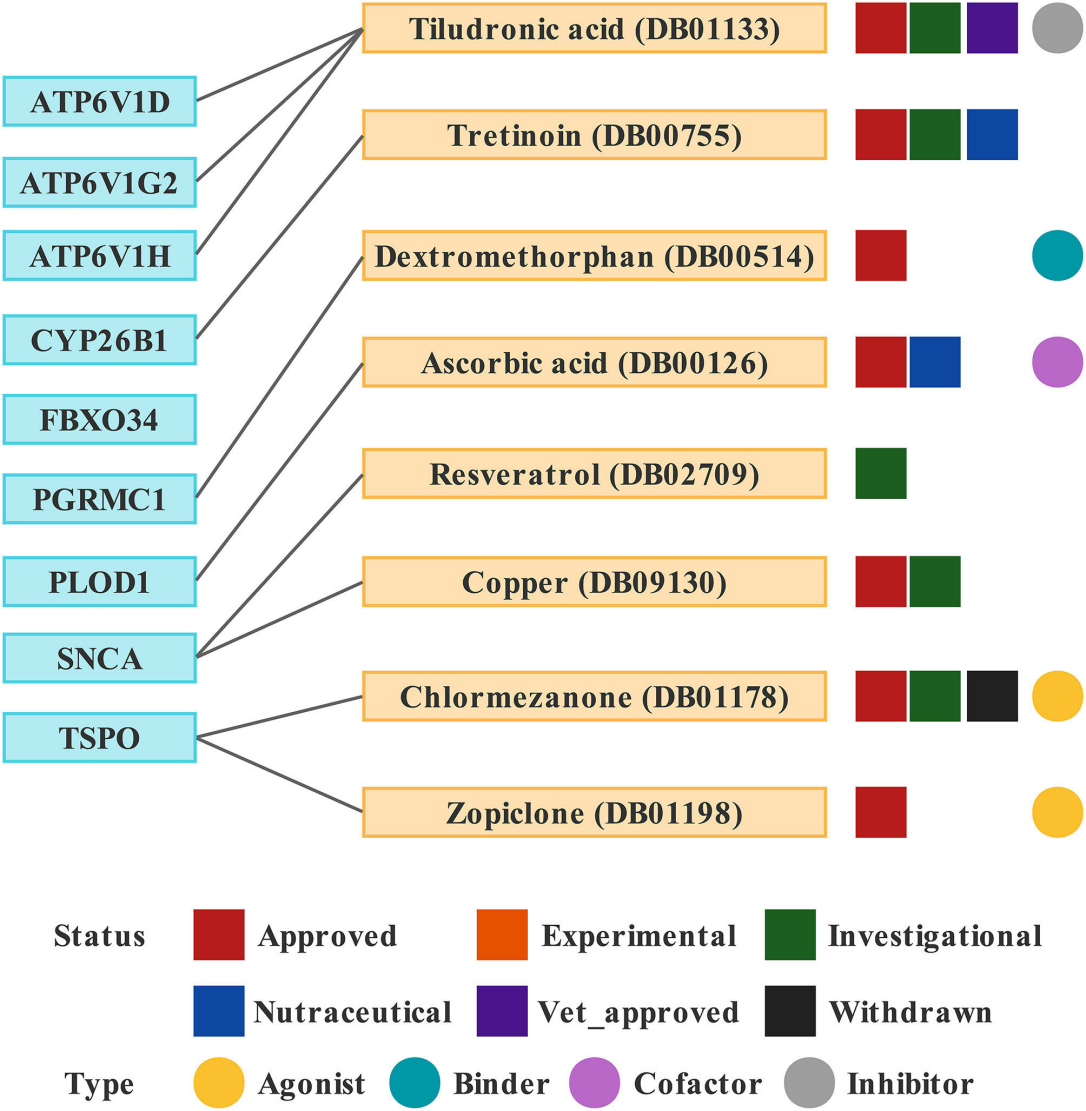
Drugs targeting these nine hub genes obtained from the DrugBank database. Drug statuses, including approved, experimental, investigational, nutraceutical, vet_approved and withdrawn, are indicated by colored squares. Drug types, including agonist, binder, cofactor, and inhibitor, are indicated by colored circles.

Based on drug and target information from the DrugBank database, eight drugs targeting these nine hub genes were identified (Figure 10). Among these drugs, six drugs were approved, one was an investigational drug, and one was withdrawn. Tiludronic acid (DB01133), an inhibitor of *ATP6V1D, ATP6V1G2*, and *ATP6V1H*, is used for the treatment of Paget's disease of the bone. Tretinoin (DB00755) targeting *CYP26B1* is used to treat fine wrinkles, acne vulgaris, and certain types of promyelocytic leukemia. Dextromethorphan (DB00514) is a binder of *PGRMC1* used to treat cases of dry cough. Ascorbic acid (DB00126) is a cofactor of *PLOD1* used to correct vitamin C deficiency and increase intestinal absorption of iron. Resveratrol (DB02709) targeting *SNCA* was investigated for the treatment of herpes labialis infections (cold sores). Both chlormezanone (DB01178) and zopiclone (DB01198) are agonists of *TSPO*. Chlormezanone is used to manage anxiety and treat muscle spasms, while zopiclone is used to treat insomnia (Supplementary Table 4).

## Discussion

Iron is involved in myelination, neurotransmitter synthesis, and mitochondrial respiration in the nervous system and could contribute to oxidative stress. Both iron overload and iron deficiency have adverse effects and may lead to neurological disorders. Relevant studies have found that increases in ferritin and iron, most likely ferritin-bound iron, are present in several brain regions involved in AD (Quintana and Gutiérrez, 2010; Galazka-Friedman et al., 2012; Raven et al., 2013).

In this study, nine genes linking AD and iron metabolism were screened. GO analysis showed that they were involved in autophagy-related and iron metabolism-related biological processes. A decrease in neuronal autophagy leads to an inability to clear pathological proteins, such as beta-amyloid (Aβ) and tau proteins (Feng et al., 2015). Several key proteins involved in the regulation of autophagy are closely associated with the development of AD (Majumder et al., 2011; Lucin et al., 2013; Heras-Sandoval et al., 2014; Sepe et al., 2014). Restoration of parkin-mediated mitophagy prevents cognitive decline (Goudarzi et al., 2021). Some neuroprotective drugs exert their effects by modulating mitophagy (Wang et al., 2018; Hirano et al., 2019; Han et al., 2020; Sun C. et al., 2020). When intracellular iron levels fall, ferritin is degraded to release free iron, a process called ferritinophagy. Biasiotto et al. proposed that abnormal ferritinophagy may be a link between impaired autophagy and dysfunctional iron homeostasis in several neurodegenerative diseases (Biasiotto et al., 2016). In addition, the GSEA showed that these hub genes were all associated with neurodegenerative diseases, especially AD, which validates the accuracy of the selection of these genes to some extent (Figure 6).

Four of the nine genes, including *ATP6V1D, ATP6V1G2, FBXO34*, and *TSPO*, were screened to construct a diagnostic model, which may be useful to guide the diagnosis of AD in clinical applications. Both *ATP6V1G2* and *ATP6V1D* encode a component of vacuolar ATPase (V-ATPase). In addition to acting as an H+ pump, V-ATPase is also involved in enzyme activity, the coupled transport of substrates across membranes, and the dissociation of ligands from receptors (Huynh and Grinstein, 2007; Vahlensieck et al., 2021). A deficiency of V-ATPase can lead to central nervous system disorders such as AD and PD (Lee et al., 2010; Williamson and Hiesinger, 2010). *ATP6V1G2* is downregulated in AD as a key metabolic gene and is involved in lysosomal transport, transporting protons from the cytoplasm to the lysosome, and maintaining lysosomal acidification (Li et al., 2020). *ATP6V1D* is associated with mitochondrial function and impaired bioenergetic metabolism (Alves et al., 2015). *FBXO34* belongs to the F-box protein family, and F-box proteins are junction proteins for Skp1-Cul1-FBP (SCF)-type E3 ubiquitin ligases, directing the ubiquitination of numerous proteins (Randle and Laman, 2016). Ubiquitinated proteins are found in neurofibrillary tangles and oligomeric Aβ plaques, and a mutation of the ubiquitin-B+1 gene leads to neuronal degeneration, which is associated with spatial reference memory impairment (van Tijn et al., 2011) and AD (Tan et al., 2007). The ubiquitin–proteasome system is also related to several neuronal signaling pathways (Zhao et al., 2003; Patrick, 2006). The ubiquitin–proteasome system and tau phosphorylation in AD are closely related (Ciechanover and Kwon, 2015; Kumar P. et al., 2015). Gong et al. found that FBXO2 ubiquitinates β-secretase, which leads to protein degradation and reduced Aβ production (Gong et al., 2016). *TSPO* is mainly found in the outer mitochondrial membrane. In response to inflammatory stimulation, *TSPO* expression is highly upregulated in various inflammatory diseases, including AD (Rupprecht et al., 2010; Selvaraj and Stocco, 2015). *TSPO* is shown to be increased in postmortem brain samples from patients with AD (Venneti et al., 2009; Gui et al., 2020), which is consistent with our study. *TSPO* deficiency does not affect Aβ production, but it does accelerate Aβ deposition, leading to more senile plaques (Zhang H. et al., 2021). Studies have shown the presence of the rs6971 single nucleotide polymorphism (SNP) in *TSPO*, which produces different radioligand binding affinities (Owen et al., 2011; Tournier et al., 2020). Currently, *TSPO* is the primary target for PET and SPECT *in vivo* neuroinflammatory imaging to monitor the inflammatory state in the brain (Tournier et al., 2020; López-Picón et al., 2022). According to one of the hypotheses of AD pathogenesis, mitochondrial dysfunction occurs in the early stages, and thus, maintaining mitochondrial function may be a therapeutic strategy for AD (Readnower et al., 2011; Eckert et al., 2012; Kumar A. et al., 2015). Kim et al. identified novel *TSPO* ligands that can restore Aβ-induced mitochondrial dysfunction and improve cognitive impairment in mouse models with AD (Kim et al., 2021).

Intrinsic immune cells such as microglia and astrocytes, as well as peripheral immune cells, are involved in the neuroinflammation associated with AD (Calsolaro and Edison, 2016; Dionisio-Santos et al., 2019; Wyatt-Johnson and Brutkiewicz, 2020; Leng and Edison, 2021). Here, immune cell infiltration and immune factors were further explored. Box plots showed significant differences in multiple immune cells between the AD and the ND groups. M1 macrophages were found to be elevated in AD, which is consistent with a previous study (Liu et al., 2022). When stimulated by pro-inflammatory factors, macrophages are converted to the M1 phenotype. Upon recognition by specific receptors, it in turn secretes other pro-inflammatory mediators that further promote the conversion of unpolarized macrophages to M1, thus forming a positive pro-inflammatory feedback loop (Gate et al., 2010; Weisser et al., 2013; Sanz et al., 2020). In addition, neutrophils and gamma delta T cells were also upregulated in AD. Increased neutrophil accumulation was found in AD brains and AD model mice, and neutrophil hyperactivity is thought to be a feature of AD (Katayama, 2020; Kong et al., 2020). Katayama found that neutrophils appear to be drivers of AD and they secrete large amounts of reactive oxygen species (Katayama, 2020). Interleukin-17 (IL-17)-producing cells, mainly γδ T cells, accumulate in the brain and meninges of women with AD, accompanied by a cognitive decline (Brigas et al., 2021). IL-17-producing cells have been identified as key players in disease progression, as they promote a local immune amplification loop in the meninges and cause disruption of the blood–brain barrier (Shichita et al., 2009; Sutton et al., 2009; Gelderblom et al., 2012; Benakis et al., 2016). Heatmaps showed a strong correlation between these hub genes and different immune factors. IL-10 single nucleotide polymorphisms were found to affect the susceptibility to AD pathology (Babić Leko et al., 2020; Yang et al., 2021). When stimulated by anti-inflammatory cytokines, macrophages are converted to the M2 phenotype. Upon recognition by specific receptors, it in turn secretes other anti-inflammatory factors that promote the conversion of unpolarized macrophages to M2, thus forming a positive feedback mechanism for the anti-inflammatory response (Gordon and Martinez, 2010; Mulder et al., 2014; Murray et al., 2014). In addition, a promising lead compound promoting IL-10 activity was provided, confirming that promoting IL-10 expression may be useful to treat AD and stroke (Sun P. et al., 2020). The interaction between astrocytic CXCL1 and neuronal CXCR2 receptors exacerbates the synaptotoxic effects of Aβ and is expected to serve as a novel target for the treatment of AD (Perez-Nievas et al., 2021). CX3CL1 was found to be decreased in the cerebrospinal fluid of patients with AD (Perea et al., 2018), which is consistent with our findings. CX3CL1 has been shown to be a strong activator of adult neurogenesis, reducing neuronal loss and improving the cognitive function of patients with AD (Fan et al., 2020). In addition, the critical roles of CX3CL1/CX3CR1 and ATP/P2X_7_R in regulating microglial activation in AD have been reviewed (Suresh et al., 2021).

Finally, eight drugs targeting the above genes were retrieved from the DrugBank database. Zopiclone (DB01198) is a non-benzodiazepine hypnotic used for the short-term treatment of insomnia. Several studies have found zopiclone to be helpful for insomnia in patients with AD, thereby improving their quality of life (Katsunuma et al., 1998; Richardson et al., 2021; Louzada et al., 2022). Tiludronic acid (DB01133), a bisphosphonate, was first described for the treatment of Paget's disease of the bone (Reginster et al., 1988). A study found that tiludronic acid and olsalazine may be potential drugs for the treatment of AD through *in silico* predictions, but further experimental validation is needed (G et al., 2020). Ascorbic acid (DB00126) is highly concentrated in the brain as a neuroprotective compound and may combat neurotoxic and neurodegenerative diseases, including AD (Moretti and Rodrigues, 2021). Jang *et al*. found that a mixture of Schisandra Chinensis extract and ascorbic acid improves mitochondrial function and memory and can also be used to alleviate AD and aging-related memory decline (Jang et al., 2020). Dextromethorphan (DB00514) is an NMDA receptor antagonist. Deuterated dextromethorphan/quinidine (AVP-786) is a promising and well-tolerated treatment option for agitation in AD, having completed two phase III trials (Garay and Grossberg, 2017; Khoury et al., 2021; Khoury, 2022). Resveratrol (DB02709), a polyphenolic phytoalexin, has been reported to affect several AD-related- and neuroprotective genes (Dennison et al., 2022). Resveratrol can improve AD-associated pathologies and produce therapeutic effects (Gomes et al., 2018; Rahman et al., 2020; Yan et al., 2020; Fang et al., 2021; Gu et al., 2021; Jang et al., 2021; Abozaid et al., 2022). Copper (DB09130) is a transition metal and a trace element in the body. Copper imbalance is associated with the pathogenesis of AD, which is an avenue for new therapeutic strategies (Ejaz et al., 2020; Zhu et al., 2020; Zubčić et al., 2020; Lei et al., 2021; Pal et al., 2021).

## Conclusion

Through bioinformatics approaches, we obtained nine hub genes linking iron metabolism and AD. The biological processes and pathways in which they are involved were explored, which will help in understanding the development of AD. However, further experimental validation is required to verify these functions. Based on the logistic regression analysis, we constructed a diagnostic model that can diagnose patients with AD by detecting the expression of several genes in the blood. In addition, these hub genes have been found to be associated with different immune factors, suggesting that they may also have an important role in the immune microenvironment. However, further studies are needed to explore their specific roles. Currently, only a few drugs targeting these hub genes are predicted to alleviate AD, suggesting that additional drugs need to be developed.

## Data Availability Statement

The original contributions presented in the study are included in the article/Supplementary Material, further inquiries can be directed to the corresponding authors.

## Author Contributions

XG, XC, and CL conceived and designed the study. DL, KC, and SL were responsible for the collection and assembly of data, data analysis, and interpretation. DL and XG were involved in the writing of the manuscript. BC, PZ, and GH provided help in revising the manuscript. All authors read and approved the final manuscript.

## Funding

This work was supported by the National Natural Science Foundation of China (81772829 and 81830052), the Special Program for Collaborative Innovation, the Construction Project of Shanghai Key Laboratory of Molecular Imaging (18DZ2260400), the Top-100 Talent Cultivation Plan of Shanghai University of Medicine and Health Sciences, Funding Scheme for Training Young Teachers in Shanghai Colleges, and Innovative Team of Intelligent Inspection and Active Health (ITIH).

## Conflict of Interest

The authors declare that the research was conducted in the absence of any commercial or financial relationships that could be construed as a potential conflict of interest.

## Publisher's Note

All claims expressed in this article are solely those of the authors and do not necessarily represent those of their affiliated organizations, or those of the publisher, the editors and the reviewers. Any product that may be evaluated in this article, or claim that may be made by its manufacturer, is not guaranteed or endorsed by the publisher.

## Supplementary Material

Supplementary Figure 1 Expression of hub genes in the AD and ND groups of blood samples from GSE63061. **(A)** ATP6V1D. **(B)** ATP6V1G2. **(C)** ATP6V1H. **(D)** CYP26B1. **(E)** FBXO34. **(F)** PGRMC1. **(G)** PLOD1. **(H)** SNCA. **(I)** TSPO.

Supplementary Figure 2 Expression of hub genes in the AD and ND groups of blood samples GSE85426. **(A)** ATP6V1D. **(B)** ATP6V1G2. **(C)** FBXO34. **(D)** PGRMC1. **(E)** PLOD1. **(F)** SNCA. **(G)** TSPO.

Supplementary Table 1 Five hundred twenty genes obtained from 14 iron metabolism-related gene sets.

Supplementary Table 2 Different immune factors were downloaded from the TISIDB database.

Supplementary Table 3 Differentially expressed genes between AD and ND samples in GSE132903.

Supplementary Table 4 Information on drugs targeting these nine hub genes.
