# Supplementary material for: Hub Genes, Diagnostic Model, and Predicted Drugs Related to Iron Metabolism in Alzheimer's Disease
Source: Front Aging Neurosci. 2022 Jul 7;14:949083. doi: 10.3389/fnagi.2022.949083 (PMC9300955; doi:10.3389/fnagi.2022.949083)
Supplement: Supplementary Table 4 — Information on drugs targeting these nine hub genes. [file Table_4.DOCX]

**Table S4:** Information on drugs targeting these nine hub genes.

| **DrugBank ID** | **Name** | **Indication/Associated Conditions** |
| --- | --- | --- |
| DB01133 | Tiludronic acid | Paget's Disease of Bone |
| DB00755 | Tretinoin | Acne Vulgaris |
|  |  | Alopecia |
|  |  | Cornification and dystrophic skin disorders |
|  |  | FAB classification M3 Acute promyelocytic leukemia |
|  |  | Fine Wrinkles |
|  |  | Skin hyperpigmentation |
|  |  | Solar Lentigines |
|  |  | Keratinization disorders of the feet |
|  |  | Keratinization disorders of the hand |
|  |  | Moderate Melasma |
|  |  | Mottled hyperpigmentation |
|  |  | Severe Melasma |
|  |  | Severe, recalcitrant Cystic acne |
|  |  | Tactile roughness of facial skin |
| DB00514 | Dextromethorphan | Allergic cough |
|  |  | Common cold |
|  |  | Common cold / flu |
|  |  | Cough |
|  |  | Cough caused by common cold |
|  |  | Cough caused by allergies |
|  |  | Cough caused by bronchitis |
|  |  | Cough caused by influenza |
|  |  | Cough caused by influenza |
|  |  | Fever |
|  |  | Influenza caused by influenza |
|  |  | Headache |
|  |  | Irritating cough |
|  |  | Itchy nose |
|  |  | Itchy throat |
|  |  | Nasal congestion |
|  |  | Pseudobulbar nasal congestion (PBA) |
|  |  | Nasal bleeding |
|  |  | Sneezing |
|  |  | Upper respiratory tract symptoms |
|  |  | Edema and itchy eyes |
| DB00126 | Ascorbic acid | Common Cold |
|  |  | Deficiency, Vitamin A |
|  |  | Deficiency, Vitamin D |
|  |  | Fever |
|  |  | Flu caused by Influenza |
|  |  | Folate deficiency |

**Table S4 (continued)**

| **DrugBank ID** | **Name** | **Indication/Associated Conditions** |
| --- | --- | --- |
| DB00126 |  | Iron Deficiency (ID) |
|  |  | Iron Deficiency Anemia (IDA) |
|  |  | Oral bacterial infection |
|  |  | Scurvy |
|  |  | Vitamin C Deficiency |
|  |  | Vitamin Deficiency |
| DB02709 | Resveratrol | Herpes labialis infections (cold sores) |
| DB09130 | Copper | the supplementation of total parenteral nutrition |
|  |  | contraception with intrauterine devices |
| DB01178 | Chlormezanone | anxiety |
|  |  | muscle spasm |
| DB01198 | Zopiclone | Insomnia |
